# Pneumoproteins in sewage workers exposed to sewage dust

**DOI:** 10.1007/s00420-012-0747-7

**Published:** 2012-02-17

**Authors:** Kari Kulvik Heldal, Lars Barregard, Per Larsson, Dag G. Ellingsen

**Affiliations:** 1 National Institute of Occupational Health, P.O. Box 8149 Dep, 0033 Oslo, Norway; 2 Department of Occupational and Environmental Medicine, Sahlgrenska University Hospital and Academy, University of Gothenburg, Gothenburg, Sweden

**Keywords:** Pneumoproteins, Sewage dust, Exposure, Bacteria, Endotoxins

## Abstract

**Purpose:**

The association between exposure to bacteria and endotoxins in sewage dust and the serum concentrations of pneumoproteins in sewage treatment plant workers were studied.

**Methods:**

Forty-four workers from eight sewage treatment plants and 38 reference workers participated in the study. Microbial aerosol was collected by personal inhalable samplers. The concentrations of bacteria and endotoxins were determined by fluorescence microscopy and the Limulus assay, respectively. Pneumoproteins (Clara cell protein: CC16, and Surfactant proteins A and D: SP-A, SP-D) were determined by ELISA in blood samples collected post-shift.

**Results:**

The exposure to dust ranged from 0.02 to 9.3 (geometric mean (GM) 0.3 mg/m^3^, of bacteria from 0.3 to 4,900 × 10^3^ (GM 27 × 10^3^) cells/m^3^ and endotoxins from 1 to 3,160 (GM 28) EU/m^3^. The exposed workers had lower CC16 [arithmetic mean (AM) 4.9 ng/ml] compared to the referents (AM 6.4 ng/ml, *p* < 0.01). No significant difference was observed for SP-D and SP-A. Exposure to bacteria was positively associated with CC16 (*p* < 0.05) and SP-D (*p* < 0.05), adjusting for possible confounders.

**Conclusions:**

This study showed that exposed workers had lower serum concentration of CC16 as compared to the referents, which may reflect a long-term effect on secretion of these pneumoproteins. The positive association between exposure to bacteria and the serum concentrations of CC16 and SP-D may be explained by a transient increased permeability of the lung–blood barrier.

## Introduction

Workers at sewage treatment plants are exposed to a complex mixture of microorganisms, microbiological components, chemicals, and gases (Melbostad et al. 1994; Douwes et al. 2001; Spaan et al. 2008). Thus, they are at risk of developing a range of adverse health effects including airway irritation and pulmonary diseases such as toxic pneumonitis (Rylander 1999; Thorn and Kerekes 2001; Thorn et al. 2002; Thorn and Beijer 2004). We have recently reported associations between exposure to endotoxin-containing dust and respiratory symptoms, such as airway irritation and cough among sewage workers (Heldal et al. 2010). Also a lower FEV_1_/FVC ratio compared to the referents was observed.

Air samples from sewage treatment plants consist mostly of bacteria, predominantly Gram-negative (Lundholm and Rylander 1983; Spaan et al. 2008). Endotoxins, cell wall components of the Gram-negative bacteria, are regarded as strong inflammatory agents. Acute non-specific inflammatory reactions with increased levels of pro-inflammatory cytokines and biomarkers in sputum, broncho-alveolar lavage (BAL), or blood serum have been shown in both experimental and epidemiological studies (Rylander and Jacobs 1997; Thorn and Rylander 1998; Thorn 2001; Heldal et al. 2003; Michel and Murdoch 2005). It has also been suggested that repeated toxic pneumonitis reactions in chronically exposed workers may result in irreversible decreased lung function and the development of chronic obstructive pulmonary disease (COPD) (Schwartz et al. 1994; Cristiani et al. 2001; Wang et al. 2003; Rylander 2006).

Clara cell protein (CC16) is a pneumoprotein secreted from Clara cells along the bronchial tree, which has an important anti-inflammatory role in the human lung (Bernard et al. 1992; Broeckaert and Bernard 2000). From the lung epithelial lining fluid (ELF), a fraction of CC16 normally passes through the lung–blood barrier into the blood stream, where it is rapidly eliminated through renal excretion (Hermans et al. 1999). Experimental and clinical studies suggest that CC16 may be a sensitive biomarker of lung injury. Increased levels of CC16 in serum may stem from increased secretion in the respiratory tract, increased leakage through the lung–blood barrier, or decreased renal clearance (Broeckaert and Bernard 2000). On the other hand, chronic exposure to cigarette smoke has been shown to damage the Clara cells, resulting in decreased CC16 in the ELF and serum (Bernard et al. 1993; Hermans and Bernard 1999).

A recent inhalation study of healthy volunteers reported higher concentrations of CC16 in serum after exposure to lipopolysaccharide (LPS), a purified derivate of endotoxins (Michel and Murdoch 2005). In contrast, a marked decrease of secretion and synthesis of CC16 was observed after LPS-induced lung inflammation in a mouse model (Arsalane et al. 2000).

Few studies of serum pneumoprotein levels have been carried out in workers occupationally exposed to endotoxin-containing dust. Higher concentrations of CC16 compared to a control group were found among sewage workers (Steiner et al. 2005), and a lower SP-A was found among asthmatic workers (Widmeier et al. 2007). However, no associations between the exposure measurements and surfactant proteins were reported (Steiner et al. 2005; Widmeier et al. 2007; Tabrizi et al. 2010; Tchopp et al. 2011).

The purpose of this study was to examine the serum levels of the pneumoproteins CC16, SP-A, and SP-D among sewage workers and to study the associations between the exposure levels and the pneumoprotein concentrations.

## Materials and methods

### Subjects

All exposed workers employed in eight municipal sewage treatment plants were invited to participate in the study (*n* = 44). Nineteen of the exposed workers were recruited from plants where sludge was dried in separate sludge driers, while 25 were recruited from plants with chemical and mechanical sewage treatment without sludge drying. The referents were office workers (*n* = 38) from compost (*n* = 28) and sewage treatment plants (*n* = 10). All invited exposed workers and referents participated in the study.

Information on smoking habits was obtained from a general questionnaire. The subjects were classified as current or former smokers. Former smokers were defined as having stopped smoking more than 12 months earlier. Atopy was defined as positive reaction to at least one of nine common respiratory allergens (birch, timothy, wormwood, mold spores, cat, dog, horse, rabbit, mites) tested by a Phadiatop test (FEIA, UniCap system, Fürst Laboratory, Norway). Background variables of the participants are shown in Table 1.

**Table 1:** Characteristics of the population

	Referents (*N* = 38)	Sewage workers (*N* = 44)
Age, AM (SD)	43 (19)	40 (11)
Men (%)	74	96
Atopy (%)	26	18
Current smokers (%)	16*	36
Amount of current smoking, cigarette/day, AM (SD)	2 (5)	4 (6)
Tobacco consumption, packyears, AM (SD)	2.3 (7)	3.9 (7)

*AM* arithmetic means, *SD* standard deviations

* *p* < 0.05

The study was approved by the Regional Medical Ethics Board. All participants were informed about the purpose of the study and gave their written informed consent.

### Exposure assessment

The sewage drying process at the plants has been described in detail previously (Heldal et al. 2010). All work operations at the sewage plants were performed indoors. The exposure was assessed by parallel sampling using two inhalable PAS 6 cassettes (Van der Wal 1983), mounted in the breathing zone of each worker. The cassettes were connected to two pumps (PS101) operated at a flow of 2.0 l/min. The sampling time was approximately 4 h. All together 44 air measurements were collected.

Aerosols for the determination of dust particles and bacteria were collected on polycarbonate filters with pore size 0.8 μm (Poretics, Osmonics, Livermore, USA), while endotoxins were collected on glass fiber filters (Whatman GF/A, Maidstone, USA). Dust mass concentrations were determined gravimetrically in a climate-controlled weighing room. The total number of bacterial cells and fungal spores was quantified by fluorescence microscopy as previously described (Heldal et al. 1996). Endotoxins were extracted (Douwes et al. 1995) and analyzed by a quantitative kinetic chromogenic Limulus amoebocyte lysate assay according to the manufacturer’s instructions (Cambrex Bio Science Walkersville, Maryland, USA). The test was done during two consecutive weeks.

### Blood sampling and analyses

Blood samples for the determination of the pneumoproteins CC16, SP-A, and SP-D were collected after at least 1 day of exposure, between 1 and 2 PM, directly after the personal exposure measurements were ended. Whole blood was collected by venipuncture in 10-ml tubes without additives (BD Diagnostic, Plymouth, UK). Serum was obtained after coagulation for 60 min at room temperature and centrifugation for 15 min at 3,000 RPM. The serum samples were then frozen in NUNC^®^ cryotubes at –25°C no more than 2 h later and kept frozen until analysis.

The concentrations of the pneumoproteins were determined at the Department of Occupational and Environmental Medicine, University of Gothenburg. CC16 was determined using the commercially available Human Clara Cell Protein ELISA kit from BioVendor (BioVendor Laboratory Medicine, Inc., Brno, CzechRepublic) according to the manufacturer’s instructions. Determination of SP-D was performed using the SP-D ELISA kit from BioVendor, according to the protocol supplied by the manufacturer. SP-A was analyzed by sandwich ELISA as described in detail previously (Ellingsen et al. 2010). In short, the primary antibody was AB3422 (Millipore, Billerica, MA, USA); the secondary antibody was HYB 238-04 (Antibody Shop, Gentofte, Denmark).

### Statistical methods

Continuous variables were log-transformed to achieve normal distribution when the skewness exceeded 2.0. Thus, the concentrations of SP-A and exposure variables were log-transformed. For log-transformed variables, the geometric mean (GM) is presented, while the arithmetic mean (AM) is otherwise used.

Parametric statistical methods were used. Student’s *t* test was used for two-group comparisons. One-way analysis of variance (ANOVA) was used when more than two groups were compared, thereafter subcommand LSD (least significant difference test) in order to separate which groups that were different from each other. Univariate associations between variables were assessed using least square regression analysis, yielding Pearson correlation coefficients (*r*_p_) as the measure of correlation. Multiple linear regression analysis (stepwise backwards procedure) was used to assess associations between dependent variables and several independent variables simultaneously. General linear models of relevant parameters were used to calculate adjusted group estimates. The level of significance was set at 0.05 (two-tailed). The statistics were calculated with SPSS 18.0.

## Results

The airborne concentrations of dust, endotoxins, and bacterial cells in the inhalable aerosol fraction collected by personal sampling are shown in Table 2. There were positive correlations between endotoxin and bacteria concentrations (*r*_p_ = 0.37, *p* < 0.05) and between endotoxin and dust concentrations (*r*_p_ = 0.47, *p* < 0.01). Fungal spores were observed only in small numbers in a few samples, and these results have therefore not been shown.

**Table 2:** The concentration of airborne contaminants in the inhalable aerosol fraction collected by personal sampling (*N* = 44)

Exposure	GM (GSD)	Median (min–max)	Percentiles	
			75th	90th
Inhalable dust (mg/m^3^)	0.31 (4.8)	0.27 (0.02–9.3)	0.76	4.41
Endotoxins (EU/m^3^)^a^	28 (7.9)	30 (1–3,160)	73	806
Bacteria (10^3^/m^3^)	27 (8.1)	19 (0.3–4,900)	67	380

*GM* geometric means, *GSD* geometric standard deviations

^a^Endotoxin containing units

The serum concentrations of the determined pneumoproteins in the exposed subjects and the referents are shown in Table 3. The mean concentration of CC16 in serum was significantly lower in the exposed subjects as compared to the referents, while the mean concentration of SP-D was lower, but not significantly. There was no statistically significant difference in the group mean concentrations of SP-A.

**Table 3:** The concentrations of pneumoproteins in sewage workers and referents

Pneumoproteins	Referents (*N* = 38)		Sewage workers (*N* = 44)		*p* value
	*n*	AM (min–max)	*n*	AM (min–max)	
SP-A (μg/ml)^a^	37	278 (0.7–2,797)	41	169 (1.7–1,000)	0.54
SP-D (ng/ml)	38	107.7 (36.2–233.7)	39	87.8 (2.7–207.3)	0.096
CC-16 (ng/ml)	38	6.4 (3.0–17.1)	43	4.9 (1.8–13.2)	0.008

*AM* arithmetic means

^a^Geometric mean for referents and workers: 64.1 and 55.8 ug/ml, respectively

The impact of potential confounders with the respect to the exposure and pneumoproteins was assessed by using the backward procedure in a multiple linear regression analysis. Being exposed (1/0), sex (1/0), age, atopy (1/0), and being a current smoker (1/0) were included as independent variables in the models. Being exposed was negatively associated with CC16 (*p* < 0.05), and being a current smoker was nearly associated (*p* = 0.07). Stratifying for being a current smoker showed that exposed smoking workers had lower serum concentration of CC16 (AM 3.9, range 1.8–6.6 ng/ml) as compared to both smoking and non-smoking referents (non-smokers: AM 6.5, range 3.0–17.1 ng/ml, *p* < 0.05 and smokers: AM 6.3, range 4.7–9.6, *p* = 0.05, respectively). Exposed smoking workers had lower but not significantly lower CC16 than non-smoking exposed workers (AM 5.4, range 2–13.2 ng/ml, *p* = 0.08). When adjusting for current smoking, the arithmetic mean concentrations of CC16 were 5.9 ng/ml in the referents and 4.9 ng/ml in the exposed workers (*p* = 0.02).

**Fig. 1:**
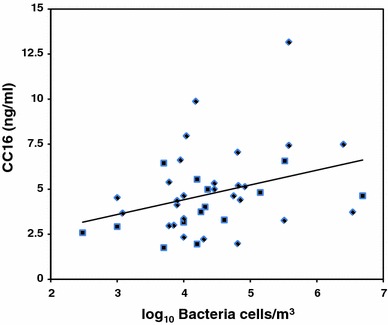
The univariate relationship between serum CC16 concentrations in 14 smoking (*filled square*) and 27 non-smoking (*filled diamond*) sewage workers and exposure to bacteria (CC16 = 3.6 + 0.8* log Bacteria cells, *R* ^2^ = 0.11, *p* < 0.05)

**Fig. 2:**
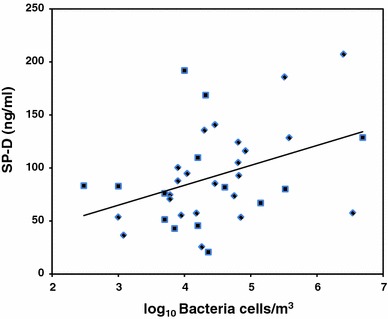
The univariate relationship between serum SP-D concentrations in 14 smoking (*filled square*) and 23 non-smoking (*filled diamond*) sewage workers and exposure to bacteria (SP-D = 8.6 + 18.8* log Bacteria cells, *R* ^2^ = 0.15, *p* < 0.05)

The associations between the pneumoprotein concentrations and the exposure to dust, bacteria, and endotoxins, respectively, were studied using regression analysis among the exposed workers only, taking into account the current smoking habits for CC16. The results showed that the concentrations of CC16 and SP-D were positively associated with the concentrations of bacteria (Table 4). The univariate relationships are illustrated in Figs. 1 and 2. No associations between any of the pneumoprotein concentrations and exposure to endotoxins or dust were observed. The spirometric lung function variables, reported previously (Heldal et al. 2010), were not significantly associated to any of the serum concentrations of the determined pneumoproteins.

**Table 4:** Results from multiple linear regression analysis assessing relation between pneumoproteins, (log) bacteria, and cigarette smoking (yes/no)

Pneumoproteins	α	β Bacteria	95%CI	β smoke	95%CI
CC-16 (ng/ml)	3.6	0.8**	0.1–1.6	−1.6^A^	−2.5 to 0.3
SP-D (ng/ml)	8.6	18.8**	3.5–34.1	−1.4^ns^	−31.5 to 28.7

Intercepts (α), regression coefficients (β), and confidence intervals (CI) are given

^A^ *p* = 0.11; ** *p* < 0.05; ^ns^ not significant

## Discussion

The results show that the mean serum concentration of CC16 was significantly lower with a tendency for SP-D in workers exposed to sewage dust as compared to the referents. However, the serum concentrations of CC16 and SP-D increased by higher personal exposure to bacterial cells sampled on the same day shortly before the collection of the blood samples. Exposure to endotoxin and dust was not associated with the pneumoproteins. No effect of exposure on the serum concentrations of SP-A was observed.

To our knowledge, pneumoprotein concentrations have only been reported in one earlier study among sewage workers. A cohort of 247 wastewater workers and 52 garbage collectors was followed up for 5 years to study respiratory health (Tchopp et al. 2011). The exposure characterization included only 11 personal exposure measurements, and only exposure to endotoxins was determined. The reported concentrations seemed to be lower than in the present study (mean 52.5 EU/m^3^, range 7.1–158 EU/m^3^) (Oppliger et al. 2005). In contrast to the present study where exposure measurements and blood sampling were performed on the same day*,* the exposure measurements were carried out at the beginning of that study. The authors concluded that exposure to organic dust containing endotoxins did not affect the lung-specific proteins, although earlier reports from the same cohort found increased serum concentrations of CC16 and lower SP-A concentrations in asthmatics (Steiner et al. 2005; Widmeier et al. 2007). This is contradictory to the findings in the present study where lower concentrations of CC16 were observed in exposed workers and no group differences were found in the SP-A concentrations.

This suggested the existence of positive associations between the exposure to bacteria and the concentrations of CC16 and SP-D, which may be explained by a temporary increased leakage into serum or an increased synthesis of these proteins. By contrast, a lower mean serum concentration of CC16 in the exposed workers as compared to the referents was observed. This could suggest a more chronic effect of exposure explained by impaired synthesis or reduced pulmonary Clara cell density. A similar pattern has been shown previously in relation to chronic and acute exposure to cigarette smoke (Bernard et al. 1993, 1997; Broeckaert and Bernard 2000). Similar reaction is observed in an animal model where the effect of chemically purified LPS from endotoxins on the level of CC16 has been studied. Pulmonary inflammation in mice, induced by intratracheal instillation of LPS, was followed by marked pulmonary decrease in the synthesis and secretion of CC16 (Arsalane et al. 2000). At the same time, a rapid increase in the serum CC16 concentrations was observed. In contrast, Michel et al. (2005) observed a dose-related increase in the serum concentrations of CC16 in healthy subjects after LPS inhalation. They suggested that the increased concentration of CC16 was caused by increased permeability of the alveolocapillary barrier.

No dose–response associations were observed between the concentrations of pneumoproteins and exposure to endotoxin or dust particles among sewage workers in this study. In general, organic dust aerosols in work environments are most often complex, containing dust particles, various microorganisms, and microbial components. A general shortcoming in many epidemiological studies is poor exposure characterizations, making it difficult to compare results across studies.

The aerosol generated from sewage may be less complex with respect to microorganisms and is thus often described as endotoxin-containing dust because of its high content of endotoxin. A few studies have also reported exposure to fungal spores and fungal cell wall constituents as well (Prażmo et al. 2003; Krajewski et al. 2004). Personal airborne exposure among sewage workers is in most studies assessed by the determination of endotoxin, only. In this study, exposure to dust particles, endotoxins, bacterial cells, and fungal spores was investigated. The exposure to endotoxins reached concentrations as high as those reported to impair lung function among cotton workers (90 EU/m^3^) (Castellan et al. 1987; DECOS 2010). The effects of exposure to bacteria in organic dust on the airways are less documented in sewage workers. The levels of bacteria were comparable to those found among sewage workers who reported irritative symptoms from the airways (Melbostad et al. 1994). However, in these workers, both the exposure to dust particles and endotoxins were associated with airway symptoms (Heldal et al. 2010). Thus, several contaminants in sewage dust may contribute to airway effects among these workers.

We have previously reported that this population of sewage workers had a poorer lung function compared to the referents (Heldal et al. 2010). However, only a minor cross-shift change in lung function parameters was observed, which may indicate that the effects were mainly chronic. It is biologically plausible that long-term exposure to sewage dust may cause damage to the Clara cells, thereby decreasing the synthesis or secretion of CC16, especially if the exposure to endotoxins is sufficiently high to affect lung function as in these sewage workers.

The mean serum concentrations of SP-A were comparable in the exposed workers and the referents. SP-A levels in serum has been reported to increase if the lung–blood barrier is affected (Hermans and Bernard 1998). However, SP-A in serum has large interindividual variability (Carbonnelle et al. 2002) and shortcomings in the analytical methods, making the results less reliable.

In conclusion, the exposed workers had lower concentrations of CC16 compared to non-exposed referents. This could suggest that long-term exposure may compromise the synthesis or secretion of the proteins. Furthermore, statistically significant associations between airborne exposure to bacteria and the serum concentrations of CC16 and SP-D, respectively, were observed. This may be explained by a transient increased leakage of these pneumoproteins through the lung–blood barrier during short-term high exposure to sewage dust.
